# Transcriptional profiling of *Pseudomonas aeruginosa* biofilm life cycle stages reveals dispersal-specific biomarkers

**DOI:** 10.3389/fmicb.2026.1826561

**Published:** 2026-04-16

**Authors:** Xavier Bertran Forga, Kathryn E. Fairfull-Smith, Jilong Qin, Makrina Totsika

**Affiliations:** 1 Centre for Immunity and Infection Control, School of Biomedical Sciences, Queensland University of Technology, Brisbane, QLD, Australia; 2 Max Planck Queensland Centre, Queensland University of Technology, Brisbane, QLD, Australia; 3 School of Chemistry and Physics, Queensland University of Technology, Brisbane, QLD, Australia; 4 Centre for Materials Science, Queensland University of Technology, Brisbane, QLD, Australia; 5 Institute for Molecular Bioscience, The University of Queensland, Brisbane, QLD, Australia

**Keywords:** biofilm dispersal, biofilm life cycle, biofilm maturation, biomarkers, c-di-GMP, dispersal biomarkers, pseudomonas aeruginosa, RNA-Seq

## Abstract

Bacteria exhibit two lifestyles: planktonic, free-floating individual cells or sessile multicellular aggregates known as biofilms. The biofilm life cycle is characterized by three distinct stages: attachment, maturation and dispersal. Consequently, specific adaptations occur at each stage, governing cellular behaviors such as adhesion and the synthesis and degradation of extracellular matrix components. Characterizing stage-specific bacterial profiles therefore represents a valuable strategy for the development of novel antibiofilm therapies. Here, we used the model biofilm-forming bacterium *Pseudomonas aeruginosa* PAO1 to characterize the transcriptional profiles of each stage of the biofilm life cycle: attachment, biofilm maturation and spontaneous dispersal in closed cultures. We report that, relative to biofilm dispersal, surface attachment coincided with the upregulation of genes comprising the Pil-Chp mechanosensory system (2.00–7.37-fold), whereas biofilm maturation was characterized by the upregulation of genes involved in Pel polysaccharide synthesis (∼2.5-fold relative to either attachment or dispersal), *siaD* and PA4396 diguanylate cyclases as well as phosphodiesterases *pipA, fimX* and PA5442 (2.08–3.73-fold relative to biofilm dispersal). In contrast with cells undergoing biofilm maturation, dispersing cells upregulated genes responsible for the biosynthesis of alginate, rhamnolipid, and extracellular nucleases (*eddA*, 3.28-fold; and *eddB*, 2.95-fold), as well as the transcriptional regulator of dispersal *amrZ* (6.27-fold). Additionally, genes involved in the biosynthesis and sensing of the dispersal signal cis-2-decenoic acid (*dspS*, 2.04-fold; and *dspI*, 5.59-fold), canonical phosphodiesterases (*nbdA*, 7.56-fold; and *rbdA*, 3.03-fold), four non-canonical HD-GYP phosphodiesterases and seven other c-di-GMP–related enzymes were also upregulated during dispersal. Altogether, this work provides benchmarking stage-specific transcriptional profiles characterizing the biofilm life cycle of *P. aerugiosa* in closed systems. Furthermore, it allowed the identification of a subset of fourteen genes as transcriptional biomarkers of dispersal, which were used to build reporter plasmids as tools to determine the onset of dispersal.

## Importance

Biofilm infections by *P. aeruginosa* are a major medical challenge due to the increased tolerance to antimicrobials displayed by bacteria living in sessile communities, which is lost upon spontaneous biofilm dispersal. Attachment, biofilm maturation and dispersal represent the main stages of a dynamic process known as the biofilm life cycle. However, the global regulatory responses governing transitions between these stages remain understudied. Here, we combine live microscopy and biomass quantification to track the progression of *P. aeruginosa* cultures through the three main stages of the biofilm life cycle. We show that cells from each stage recapitulate canonical, stage-specific transcriptional responses and identify a set of biomarkers associated with the onset of dispersal. These biomarkers may offer a practical tool for rapidly screening dispersal-inducing compounds, aiding in the discovery of the next generation of antibiofilm therapeutics.

## Introduction

*Pseudomonas aeruginosa* is a highly adaptable Gram-negative opportunistic pathogen, which survives in hostile environments by forming cell aggregates embedded in a protective extracellular matrix primarily composed of exopolysaccharides, extracellular DNA (eDNA) and structural proteins (Flemming and Wingender, 2010). These aggregates, known as biofilms, exhibit enhanced tolerance to environmental stresses, including desiccation, host defenses, and clinically relevant antimicrobial agents, compared to their planktonic counterparts (Høiby et al., 2008). In clinical settings, this heightened tolerance enables biofilm-associated *P. aeruginosa* populations to persist under hostile conditions and establish highly recalcitrant chronic infections (Donlan and Costerton, 2002).

In laboratories, *P. aeruginosa* biofilms are frequently studied using a variety of culture systems such as closed culture systems (e.g. microtiter plates) or open-flow systems (e.g., flow cells) (Irie and Parsek, 2014; O’Toole, 2011; Pamp et al., 2008). The latter, which allow the continuous influx and efflux of media to maintain culture homeostasis, have been extensively used to identify and study the three main stages of the biofilm life cycle (attachment, biofilm maturation and dispersal), as well as distinct stage-specific molecular mechanisms (Sauer et al., 2002; Southey-Pillig et al., 2005). However, whether these stages are effectively reproduced in closed culture systems remains understudied. During attachment, cells transition from the motile to the sessile lifestyle. This process is mediated by the chemotaxis systems Pil-Chp and Wsp. Pil-Chp is a type IV pili-associated system that senses pilus tension upon surface contact (Inclan et al., 2016; Persat et al., 2015). In contrast, Wsp is proposed to sense physical perturbations in the cell envelope (O’Connor et al., 2012; O’Neal et al., 2022). Upon their activation, both systems transduce signals that directly (Wsp via the associated WspR) or indirectly (Pil-Chp through activation of cAMP-associated pathways) promote the synthesis of the secondary messenger cyclic-di-GMP (c-di-GMP) (O’Toole, 2011; Park and Sauer, 2022), The concentration of c-di-GMP is controlled by a family of proteins harboring diguanylate cyclase domains (DGCs; possessing a GGDEF motif) and phosphodiesterase domains (PDE; possessing HD-GYP or EAL motifs), which respectively synthesize and degrade it (Hengge, 2016; Hengge, 2021; Povolotsky and Hengge, 2012; Randall et al., 2022).

The accumulation of c-di-GMP in turn mediates the upregulation of genes involved in the synthesis of matrix components and fimbrial adhesins such as CupA (Güvener and Harwood, 2007; Patino et al., 2025; Vallet et al., 2001). During biofilm maturation, the components of the matrix are progressively secreted into the extracellular space and cells become densely packed to form defined microcolonies (Purevdorj-Gage et al., 2005). Subsequently, environmental cues and native intracellular signals may accumulate, such as quorum sensing signals mediating the synthesis of rhamnolipids or the fatty acid dispersal signal cis-2-decenoic acid, eventually triggering spontaneous dispersal responses (Boles et al., 2005; McDougald et al., 2012). This phenomenon is characterized by biofilm-residing single cells regaining motility and resuming the planktonic lifestyle, during which they escape the biofilm matrix by hydrolyzing its components (Rumbaugh and Sauer, 2020). This is achieved via the expression of glycosyl hydrolases such as PslG and PelA that degrade matrix exopolysaccharides Psl and Pel degradation (Cherny and Sauer, 2019; Cherny and Sauer, 2020; Rumbaugh and Sauer, 2020), and extracellular endonucleases EndA, EddA, and EddB (Cherny and Sauer, 2019; Cherny and Sauer, 2020; Rumbaugh and Sauer, 2020).

The transition between the stages of the biofilm life cycle is driven by native signaling pathways. Nevertheless, studies characterizing the pathways involved in the dispersal stage have applied transcriptomics on biofilms treated with biofilm-dispersing agents such as NO donors (e.g., SNP) or carbon sources (e.g., glutamate), which captured immediate transcriptional changes derived from an abrupt interruption of the biofilm maturation process (Chua et al., 2014; Kalia and Sauer, 2024; Zhu et al., 2019). However, the progression through the biofilm stages is a dynamic process (An et al., 2010; Bertran et al., 2025), urging the temporal resolution of stage-specific gene expression. Therefore, we here performed RNA sequencing to compare the currently understudied transcriptional variations across cells undergoing attachment, biofilm maturation, and dispersal in closed systems. Subsequently, by tracking the transcription patterns of genes throughout the three life cycle stages, we identified a subset of biomarkers for biofilm dispersal, which were used to build a collection of reporter plasmids as screening tools for the identification of the onset of dispersal.

## Results

### *P. aeruginosa* biofilms cultured in closed systems recapitulate the three defined life stages of open-flow biofilms

**FIGURE 1:**
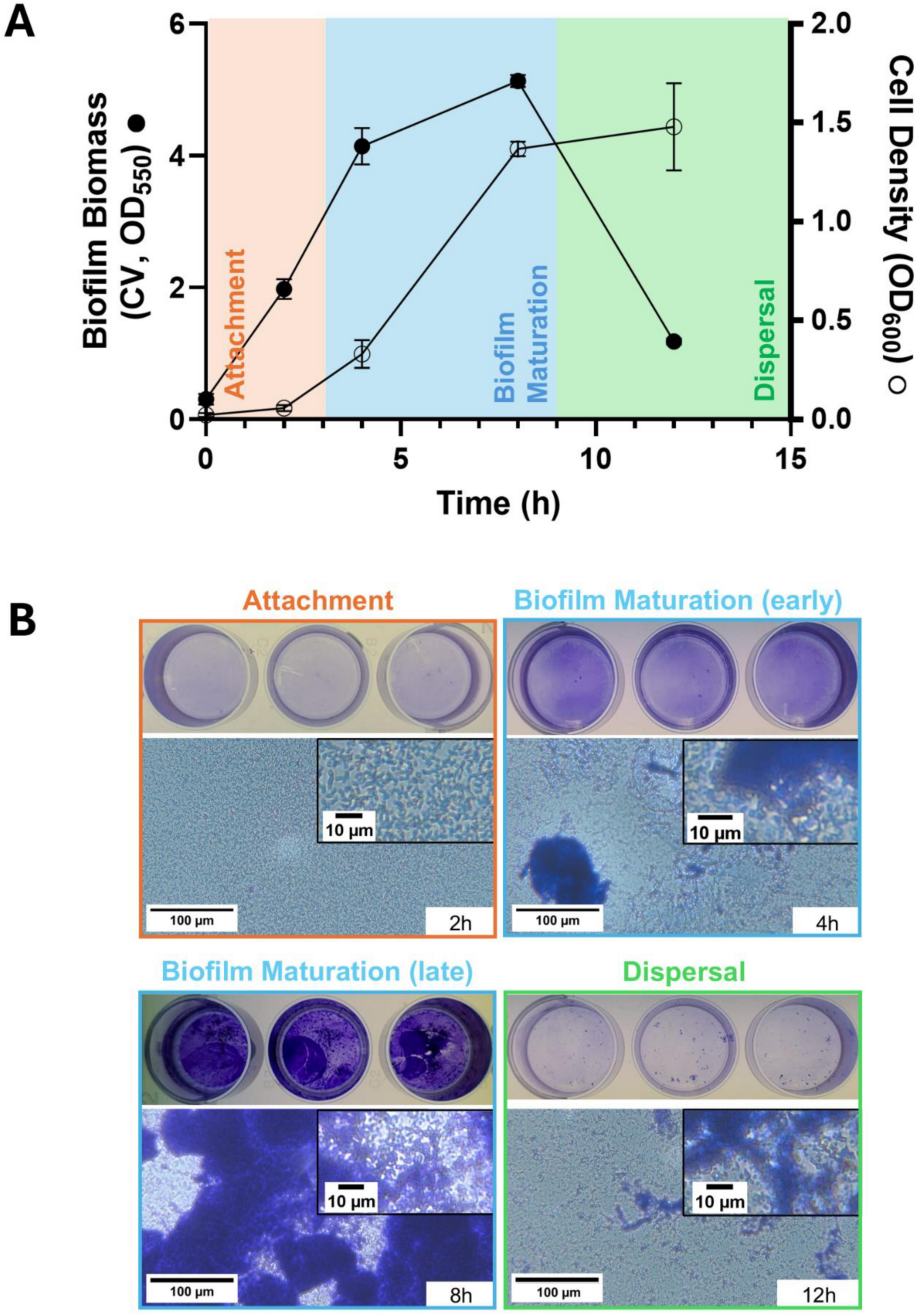
Biofilm formation and dispersal kinetics of *P. aeruginosa* PAO1. **(A)** *P. aeruginosa* PAO1 biofilm biomass and culture turbidity over 12 h. PAO1 cultures were seeded on 24-well plates and biofilm biomass was quantified at different time points by crystal violet (CV) staining (●). Culture density was simultaneously monitored by OD_600_ (○) with means ± SD shown. 3 biological replicates are represented. **(B)** Micrographs of the crystal violet-stained biofilms at each stage of the biofilm life cycle. Crystal-violet-stained microscopic images of *P. aeruginosa* biofilm cultures in 24-wells. Images are representative of at least 3 biological replicates. Orange: attachment stage. Blue: biofilm maturation stage. Green: dispersal stage. This color coding scheme is used throughout the article to differentiate the three biofilm stages.

We previously reported that the biofilm growth kinetics of the model organism *P. aeruginosa* PAO1 in closed cultures displayed progressive accumulation of surface-attached biomass, reaching maximum biomass between 2 and 8 h, followed by a marked reduction (Bertran et al., 2025). While the stages of the biofilm life cycle (attachment, maturation and dispersal) have been primarily described in open-flow cultures, these remain understudied in closed culture systems such as microtiter plates. To further characterize how these stages manifest in closed culture systems, we quantified the biofilm biomass via crystal violet staining on forming and dispersing biofilms at different time points (Figure 1A), which we combined with microscopy to capture stage-specific morphological features using microtiter plates (Figure 1B and Supplementary Figure 1). Using this approach, we observed that planktonic cells initiate attachment within 2 h of inoculation (Figures 1A,B) (orange), during which cells adhered to the surface as a monolayer, with a culture density (OD_600_) of ∼0.1 and a total biomass of ∼2 (stained by crystal violet with OD_550_). At 4–8 h post-inoculation, cells transitioned to the biofilm maturation stage (Figures 1A,B) (blue), organized in voluminous scattered clusters that grew over time and produced multicellular biofilm structures (Figure 1B and Supplementary Figure 1), as observed by peak biofilm biomass staining at the 8 h time point (OD_550_ of ∼5) (Figure 1A), which is consistent with other studies reporting biofilm biomass of *P. aeruginosa* PAO1 in this time window and similar culture conditions (Bertran et al., 2025; Forga et al., 2026; Zhu et al., 2018; Zhu et al., 2019) This build-up in biomass occurred between the early (OD_600_ ∼0.3) and the late-logarithmic phases (OD_600_ ∼1.5). Beyond 8 h post-inoculation and at a higher culture density (OD_600_ > 1.5), biofilm biomass rapidly decreased. This phenomenon was characterized by disassembled structures leaving clearly distinguishable biofilm remnants attached to the well surface and a loss of 77% of accumulated biomass (Figures 1A,B and Supplementary Figure 1) (green). Collectively, our data indicate that cells grown in closed culture systems synchronously progress through distinctive phases culminating in spontaneous (endogenously induced) biofilm dispersal at defined time-points. Therefore, closed biofilm systems can effectively recapitulate the stages of the biofilm life cycle originally described in open-flow culture systems in comparatively shorter time courses (An et al., 2010; Hsu et al., 2008; Sauer et al., 2002).

### Transcriptional profiling of *P. aeruginosa* biofilm cultures displays canonical pathways of attachment, maturation and dispersal

While *P. aeruginosa* cells grown in microtiter plates undergo the three canonical stages of the biofilm life cycle, RNA recovered from biofilm cells cultured in microtiter plates is typically insufficient in quantity to support downstream RNA sequencing (RNA-seq). Therefore, we replicated the biofilm kinetics in tissue culture flasks as a larger-volume closed culture system, monitoring biofilm cell density in addition to total biomass via staining with crystal violet. Biofilm kinetics displayed by *P. aeruginosa* PAO1 in tissue culture flasks mimicked the temporal pattern of microtiter plates in total biomass and microscopically (Supplementary Figure 1), showing attachment, biofilm maturation and dispersal within the same time frames.

**FIGURE 2:**
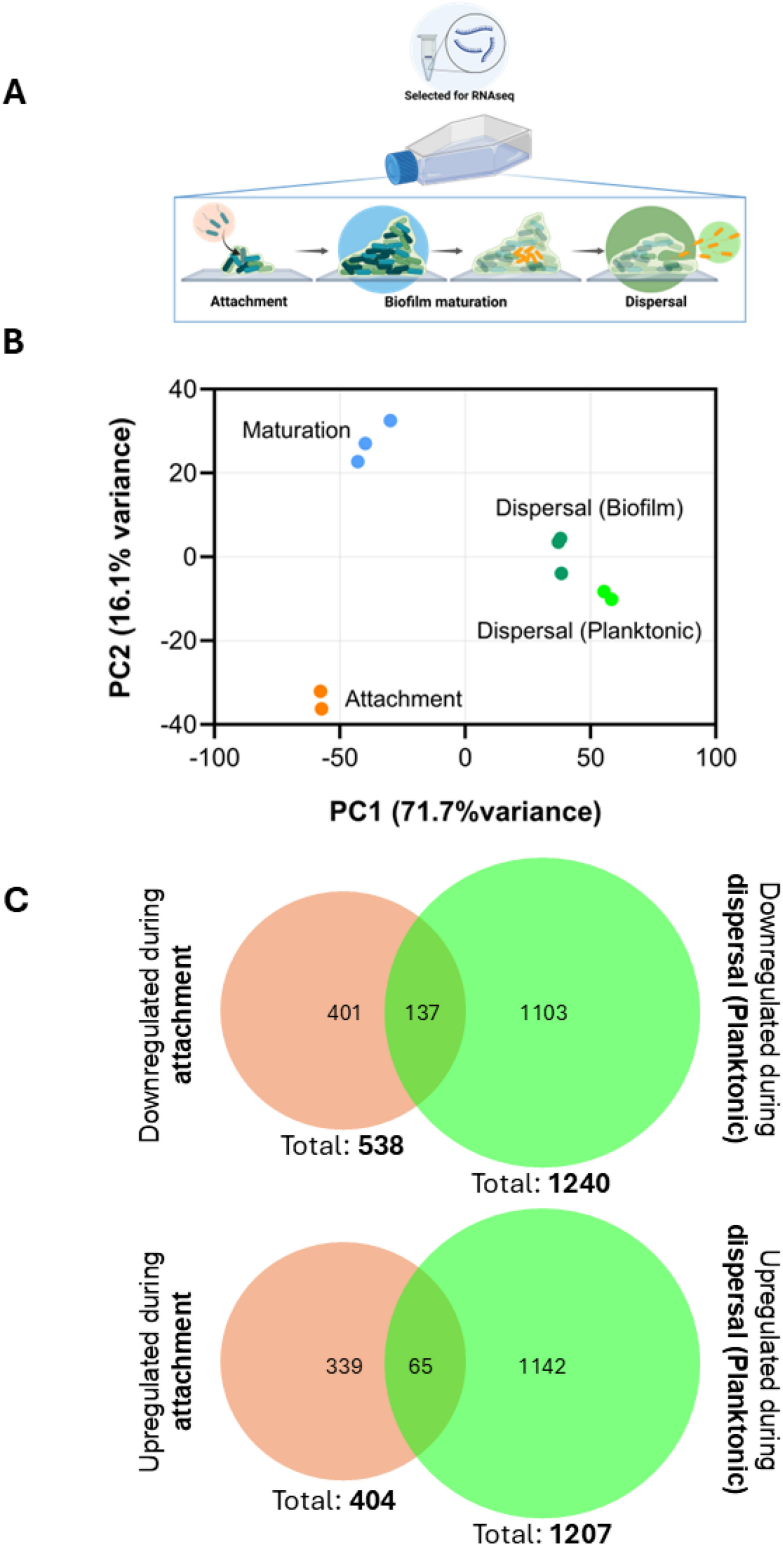
Transcriptional profiles of different stages of the biofilm life cycle. **(A)** Schematic depicting RNA sample collection for sequencing. During attachment, the free-swimming phase was collected to represent cells rapidly transitioning from motile to sessile. During biofilm maturation, surface-attached cells were collected, and cells from both phases were collected during the dispersal stage. **(B)** Principal component analysis of samples collected at distinct stages of the biofilm life cycle (attachment—orange; biofilm maturation—blue; the planktonic phase of biofilm dispersal—light green; and the biofilm phase of biofilm dispersal—dark green). Each biological replicate of a group is represented as a dot of the same color. The normalized reads of all genes were then extracted for each sample and transformed into PC loadings with ClustVis (Metsalu and Vilo, 2015). Values for PC1 and PC2, accounting for the largest variance percentage are represented in the plot. **(C)** Venn diagrams showing differentially transcribed genes in cells collected from the liquid phase undergoing attachment and dispersal (relative to biofilm maturation). Downregulated (top) and upregulated (bottom) genes at each stage relative to biofilm maturation are represented separately. Overlapping areas indicate genes with consistent transcriptional changes relative to biofilm maturation stage.

Using the flask culture system, we performed RNA sequencing of *P. aeruginosa* during attachment, early biofilm maturation and dispersal to characterize stage-specific transcriptional profiles (Figure 2A). This study design enabled temporal resolution of both global and individual gene transcription variation across the biofilm life cycle stages. Accordingly, biofilm maturation was used as the central reference point, as it represents the intermediate stage between attachment and dispersal (Figure 2C). Differential transcription analysis using a negative binomial test (*P* ≤ 0.01; Log_2_ fold-change ≥ | 1|) identified a total of 3,361 genes with significant transcriptional changes across all three comparisons: (i) Attachment vs. Biofilm Maturation; (ii) Biofilm Maturation vs. Dispersal (Planktonic); and (iii) Attachment vs. Dispersal (Supplementary Table 1). Cells collected from the liquid phase and from biofilms during the dispersal stage showed few transcriptional differences, with only 83 differentially regulated genes between the two states, and with low fold changes (Supplementary Table 1). This suggested that cells at the dispersal stage measured in this study synchronously altered their transcriptomic profiles regardless of remaining surface-associated or becoming free-swimming. Because the planktonic phase of dispersal exhibited greater transcriptional changes than the biofilm phase relative to the other groups (Figure 2B), we used it for all subsequent analyses. Principal component analysis (PCA) further confirmed that cells at each stage formed distinct clusters, indicating that distinct transcriptional profiles were associated with attachment, maturation, and dispersal (Figure 2B).

Building on these global transcriptional variations and to further validate our datasets, we mined our RNA-seq data for previously reported stage-specific changes in the transcription of genes previously associated with attachment, biofilm formation or dispersal. During attachment and extending into biofilm maturation, we observed the upregulation of the Pil-Chp surface-sensing complex known to mediate attachment (Patino et al., 2025), whereas no upregulation of the complementary Wsp system was noted. Notably, transcription of *pilA, pilB, pilD*, and *pilGHIJK-chpABCDE*, encoding type IV pili structural and signal transduction proteins, and of *cyaB* and *vfr*, which encode an adenylate cyclase and its associated cyclic-AMP-controlled transcriptional regulator Vfr, was upregulated between 2.00- and 7.37-fold relative to biofilm dispersal.

**FIGURE 3:**
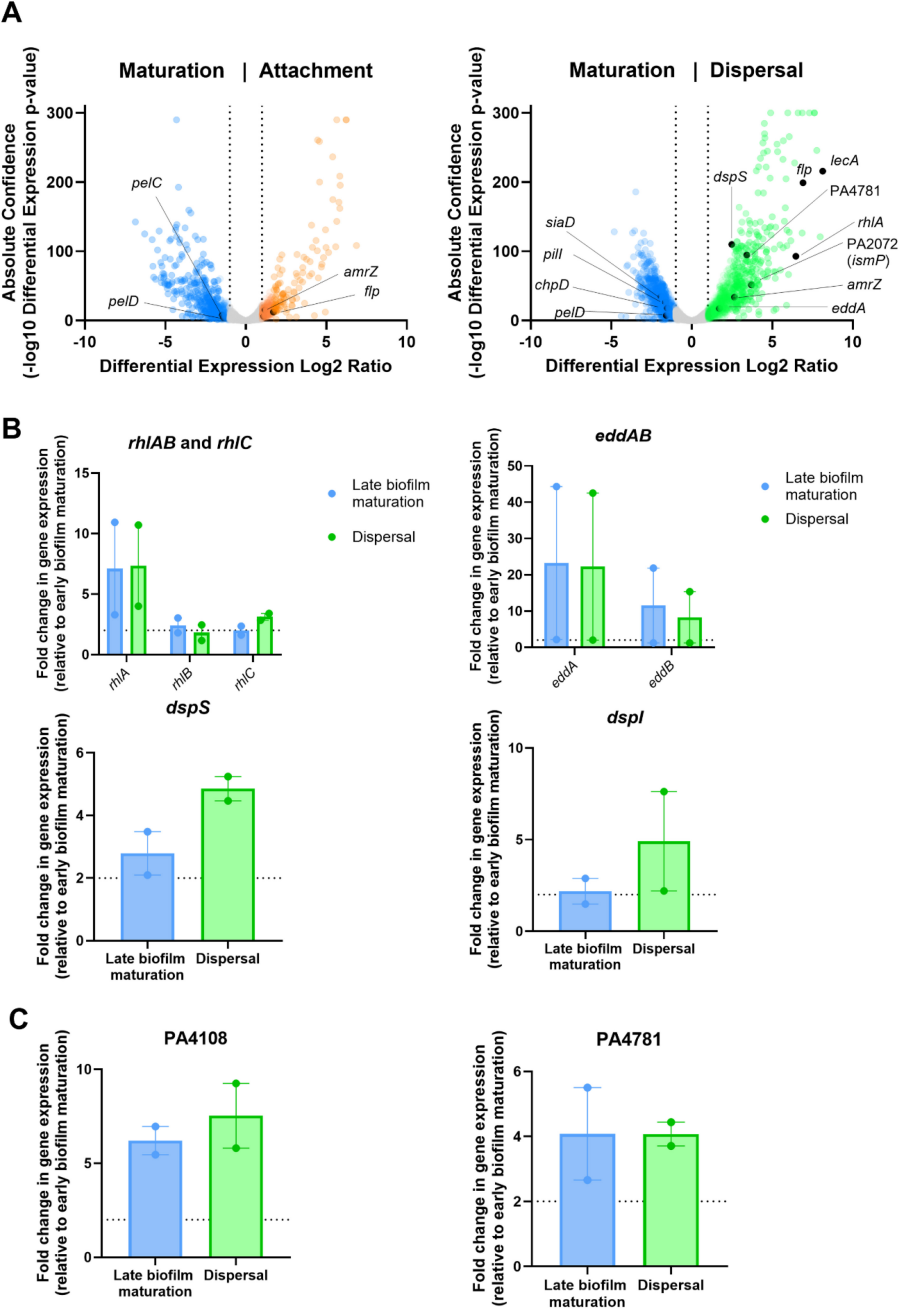
Genes involved in dispersal mechanisms are upregulated during late biofilm maturation and dispersal compared to early biofilm maturation. **(A)** Comparison of the transcriptomes of planktonic cells during attachment or dispersal to that of sessile cells during biofilm maturation. Genes containing Log2-fold change transcription values > |1| were highlighted in biofilm stage specific colors (attachment—orange; biofilm maturation—blue; biofilm dispersal –green). **(B,C)** RT-qPCR analysis of canonical dispersal genes (*rhlA, rhlB, rhlC, eddA, eddB, dspS*, and *dspI*) and the PDEs PA4108 and PA4781, which were identified as putatively involved in dispersal. RNA samples were extracted at different time points with Means ± SEM shown.

Biofilm maturation involves synthesis pathways for extracellular polysaccharides and eDNA, which are the main components of the biofilm matrix (Flemming and Wingender, 2010). During biofilm maturation, we observed the upregulation of the exopolysaccharide Pel synthesis operon *pelBCDEF* but not *pelG* relative to attachment (2.22–2.26-fold) and dispersal stages (2.16–2.99-fold) (Figure 3A and Supplementary Table 1; Jennings et al., 2015). Additionally, the alternative respiration process of denitrification is known to be active during biofilm maturation due to cell layering inside the biofilm restricting oxygen availability (Barraud et al., 2006). Accordingly, we found biofilm cells to upregulate the transcription of the nitrite reductase complex (*nirSMCFDLG*) by up to 10-fold relative to attachment.

Planktonic cells at the biofilm dispersal stage, relative to attachment and biofilm maturation stages, showed an increased transcription of genes involved in eDNA hydrolysis (*eddA*, 3.28-fold; and *eddB*, 2.95-fold; but not *endA*) (Figure 3A and Supplementary Table 1), suggesting the remodeling of the extracellular matrix to allow the escape of planktonic cells. We also observed a pronounced upregulation of quorum-sensing–regulated genes previously shown to drive biofilm dispersal. *rhlA*, *rhlB*, and *rhlC*, which encode enzymes for the biosynthesis of the rhamnolipid biosurfactant implicated in quorum-sensing–mediated dispersal were upregulated by 88-, 45-, and 12-fold, respectively (Figure 3A and Supplementary Table 1; Bhattacharjee et al., 2016; Davey et al., 2003; Glick et al., 2010; Schooling et al., 2004). Another quorum-sensing pathway activated during dispersal involved the fatty acid signal cis-2-decenoic acid (Davey et al., 2003; Kalia et al., 2023). This fatty acid signal is produced and sensed by the proteins encoded by *dspI* and *dspS*, which were upregulated by 2.04- and 5.59-fold, respectively, during dispersal relative to the biofilm maturation stage (Figure 3A and Supplementary Table 1). Interestingly, genes previously associated with biofilm maturation were also found upregulated during the dispersal stage. Adhesion factors, such as lectins *lecA* and *lecB* were highly upregulated during dispersal by 279- and 115-fold, respectively, relative to the biofilm maturation stage. Similarly, *flp*, which encodes the main subunit of the type IVb pili adhesin was upregulated by ∼120-fold, together with several alginate biosynthesis genes (*alg8*, 2.26-fold; *algX*, 2.16-fold; *algJ*, 2.39-fold; *algA*, 2.74-fold) and their associated regulators *algU* (2.49-fold), *algR* (5.76-fold) and *amrZ* (6.27-fold) (Figure 3A and Supplementary Table 1; Bernard et al., 2009; Böhning et al., 2023; Giraud et al., 2011). These findings were consistent with previous studies showing that dispersed cells had significantly increased functional adhesion relative to planktonic cells (Chambers et al., 2017). Therefore, our RNA-seq data captured transcriptional changes associated with previously documented attachment, biofilm maturation and dispersal responses, further underscoring the consistency of our dataset with established biofilm regulatory pathways and the robustness of our analytical methods.

To further validate the upregulation of these known effectors of biofilm dispersal, we extracted RNA from biofilms at 4 h (early maturation), 8 h (late maturation), and planktonic cells collected at 12 h (dispersal) and measured the transcript levels of representative genes involved in rhamnolipid synthesis (*rhlAB, rhlC*), eDNA degradation (*eddA, eddB*), and cis-2-decenoic acid signaling (*dspI, dspS*) by RT-qPCR (Figure 3B). Relative to maturing biofilms, all genes were upregulated in mature and dispersed biofilms. These findings further validate our RNA-seq data and indicate that mature biofilms may begin to transcriptionally activate dispersal pathways before the onset of active detachment.

### Specific c-di-GMP enzymes characterize the biofilm maturation and dispersal transcriptomes

Cyclic di-GMP (c-di-GMP) is a secondary signaling molecule governing the transition from biofilm maturation to dispersal. Considering that the roles of enzymes responsible for its synthesis and breakdown are stage-specific, we also analyzed our transcriptomic data to identify genes encoding enzymes with predicted PDE (EAL or HD-GYP) or DGC (GGDEF) domains (Table 1). Expectedly, genes encoding proteins essential for biofilm development (*siaD*, 3.73-fold and *pipA*, 2.08-fold) were upregulated during biofilm maturation (Cai et al., 2022; Eilers et al., 2022; Eilers et al., 2024; Wei et al., 2019). Genes implicated in the formation of large microcolonies (Eilers et al., 2022), including PA4396 (2.08-fold), *fimX* (2.77-fold), and PA5442 (2.36-fold) were also significantly upregulated. In contrast, we observed an upregulation of a combination of PDEs and DGCs in cells at the dispersal stage, including sensory transducers of dispersal cues *nbdA* (7.56-fold) and *nicD* (6.73-fold) (Cai et al., 2020; Li et al., 2013; Liu et al., 2022), as well as RbdA, a BdlA-associated PDE involved in central dispersal responses (An et al., 2010; Li et al., 2014; Petrova and Sauer, 2012a). Additionally, upregulation of *ismP* (12.99-fold) was noted, which encodes an iron sensor that under low iron conditions hijacks and inhibits a cognate DGC ImcA (Zhan et al., 2024).

**TABLE 1:** Differentially regulated genes encoding c-di-GMP-modifying enzymes during biofilm maturation and dispersal.

Locus tag	Gene name	Domain(s)	Activity	Fold-change
Biofilm maturation (vs. dispersal)				
PA0169	*siaD*	GGDEF	DGC	3.73
PA0285	*pipA*	GGDEF-EAL	PDE	2.08
PA4396	–	GGDEF	–	2.08
PA4959	*fimX*	GGDEF-EAL	PDE	2.77
PA5442	–	GGDEF-EAL	PDE	2.36
Biofilm dispersal (vs. maturation)				
PA0290	–	GGDEF	–	2.73
PA0575	*rmcA*	GGDEF-EAL	PDE	3.51
PA0847	–	GGDEF	DGC	2.09
PA0861	*rbdA*	GGDEF-EAL	PDE	3.03
PA1878	–	HD-GYP	–	3.39
PA2072	*ismP*	GGDEF-EAL	–	12.99
PA2572	–	HD-GYP	–	8.22
PA2771	–	GGDEF	DGC	2.75
PA3311	*nbdA*	GGDEF-EAL	PDE	7.56
PA3825	–	EAL	PDE	1.25
PA4108	–	HD-GYP	PDE	5.60
PA4781	–	HD-GYP	PDE	10.70
PA4929	*nicD*	GGDEF	DGC	6.73

Interestingly, dispersed cells upregulated all four genes encoding proteins with HD-GYP phosphodiesterase domains present in the *P. aeruginosa* PAO1 genome (PA1878, PA2572, PA4108, and PA4781) (Table 1). Two of these, PA4108 and PA4781 were previously reported to have PDE activity and to be necessary for swarming and the production of virulence factors (Ryan et al., 2009; Stelitano et al., 2013). Due to the known role of rhamnolipids, which are required for swarming motility and dispersal in *P. aeruginosa* (Boles et al., 2005; Davey et al., 2003; Schooling et al., 2004), we further validated the transcriptional changes of these two genes using RT-qPCR (Figure 3C), confirming an increase in expression of 7.5- and 4-fold, respectively, during dispersal relative to maturing biofilms at 4 h. Moreover, the transcription of both genes was also upregulated in mature biofilms (6.2- and 4-fold) (Figure 3C), indicating a similar pattern of transcriptional priming prior to dispersal. Altogether, our results indicate that canonical stage-specific c-di-GMP modulation responses are accurately reflected in our transcriptomic dataset and suggest a potential undescribed role of HD-GYP-containing PDEs in mediating biofilm dispersal.

### Transcriptional biomarkers identify the onset of biofilm dispersal

Since the obtained transcriptomic profiles recapitulated canonical pathways associated with attachment, maturation, and dispersal, our datasets appeared well suited to identify gene changes associated with the dispersal phase of *P. aeruginosa* and serve as transcriptional biomarkers of biofilm dispersal. We reasoned that genes directly involved in biofilm dispersal would exhibit a distinct temporal pattern of transcription such as an inverse regulation between the biofilm maturation and dispersal stages relative to the initial attachment stage. Thus, pro-dispersal genes would be repressed during maturation but strongly upregulated during dispersal, or vice versa. To identify candidate biomarkers, we applied these selection criteria and identified 14 upregulated and 8 downregulated genes that could potentially serve as dispersal biomarkers (Table 2).

**TABLE 2:** Genes differentially regulated at the biofilm maturation and dispersal stage compared to the attachment stage.

Gene	Fold change in biofilm maturation ^a^	Fold change in biofilm dispersal ^a^	Gene product
Downregulated at 4 h and upregulated at 8 h			
PA0111	−2.01	12.21	hypothetical protein
*cheR*2	−2.22	2.10	chemotaxis protein methyltransferase
PA0743	−2.58	3.12	NAD-dependent L-serine dehydrogenase
PA1353	−2.36	3.32	hypothetical protein
*pqqA*	−5.10	2.55	coenzyme PQQ synthesis protein A
PA2588	−2.03	3.01	transcriptional regulator
*amrZ*	−2.30	2.55	alginate and motility regulator Z
*tadA*	−2.17	13.64	ATPase TadA
*rcpA*	−2.13	12.47	type II/III secretion system protein
*rcpC*	−2.07	13.83	hypothetical protein
*Flp*	−3.27	34.54	type IVb pilin Flp
PA4523	−2.31	3.41	hypothetical protein
*cupE1*	−2.11	9.58	fimbrial subunit CupE1
*cupE2*	−2.04	3.95	fimbrial subunit CupE2
Upregulated at 4 h and downregulated at 8 h			
PA0485	2.93	− 2.91	hypothetical protein
PA1170	2.43	−4.76	hypothetical protein
*metH*	2.45	−2.13	B12-dependent methionine synthase
*ilvH*	3.53	−2.35	acetolactate synthase small subunit
*ilvI*	4.69	−2.57	acetolactate synthase 3 catalytic subunit
*cysN*	2.1	−2.62	bifunctional sulfate adenylyltransferase subunit1/adenylylsulfate kinase
PA4889	2.06	−14.92	oxidoreductase
PA4907	2.41	−2.27	short-chain dehydrogenase

^a^ Relative to samples collected during attachment.

Among the upregulated genes relative to cells undergoing attachment, *cheR2* (2.1-fold) (Table 2) encodes a methyltransferase component of the Che2 chemotaxis pathway, which interacts with the heme-containing oxygen sensor McbP/Aer2 and the coupling protein CheW2 to modulate virulence and chemotaxis through a cryptic signaling output (Matilla et al., 2022; Tchigvintsev et al., 2012). Another notable gene, *pqqA* (2.55-fold) (Table 2), is part of the *pqqABCDE* operon responsible for the biosynthesis of pyrroloquinoline quinone (PQQ), a redox cofactor for the quinoprotein ethanol dehydrogenases ExaA and ExaBC (Gliese et al., 2004). In addition, several genes under the transcriptional control of the two-component system PprAB were also upregulated. These include *tadA*, *rcpA*, *rcpC*, and *flp* (13.66-, 12.47-, 13.83-, and 34.54-fold) (Table 2) which are part of a contiguous gene cluster spanning PA4297 (*tadG*) to PA4306 (*flp*). This locus encodes the machinery for the synthesis and secretion of Flp type IVb pili (Tassinari et al., 2023). Notably, two of the dispersal biomarkers *amrZ* and PA2588 (*cdpR*) possess known roles in modulating biofilm physiology. *amrZ* encodes a recognized transcriptional regulator of dispersal and *cdpR* is a quorum sensing and virulence transcriptional regulator (Kalia et al., 2022; Zhao et al., 2016; Zhao et al., 2016).

Several uncharacterized genes, including PA0111, PA0743, and PA1353 were also identified as upregulated biomarkers of biofilm dispersal, many of which appear to be associated with redox processes. While no experimental data are available for PA0111, bioinformatic predictions suggest that the directly upstream gene PA0110 encodes a protein putatively related to a mitochondrial cytochrome *c* oxidase. Additionally, PA0112 and PA0113 are predicted to encode a heme A synthase and a protoheme IX farnesyltransferase, respectively, implicating these loci in cytochrome maturation or electron transport. Similarly, PA0743, which encodes an L-serine dehydrogenase, was also upregulated, potentially reflecting altered amino acid catabolism related to redox homeostasis during biofilm dispersal. No functional predictions are currently available for PA1353, which was also part of the identified dispersal biomarkers.

**FIGURE 4:**
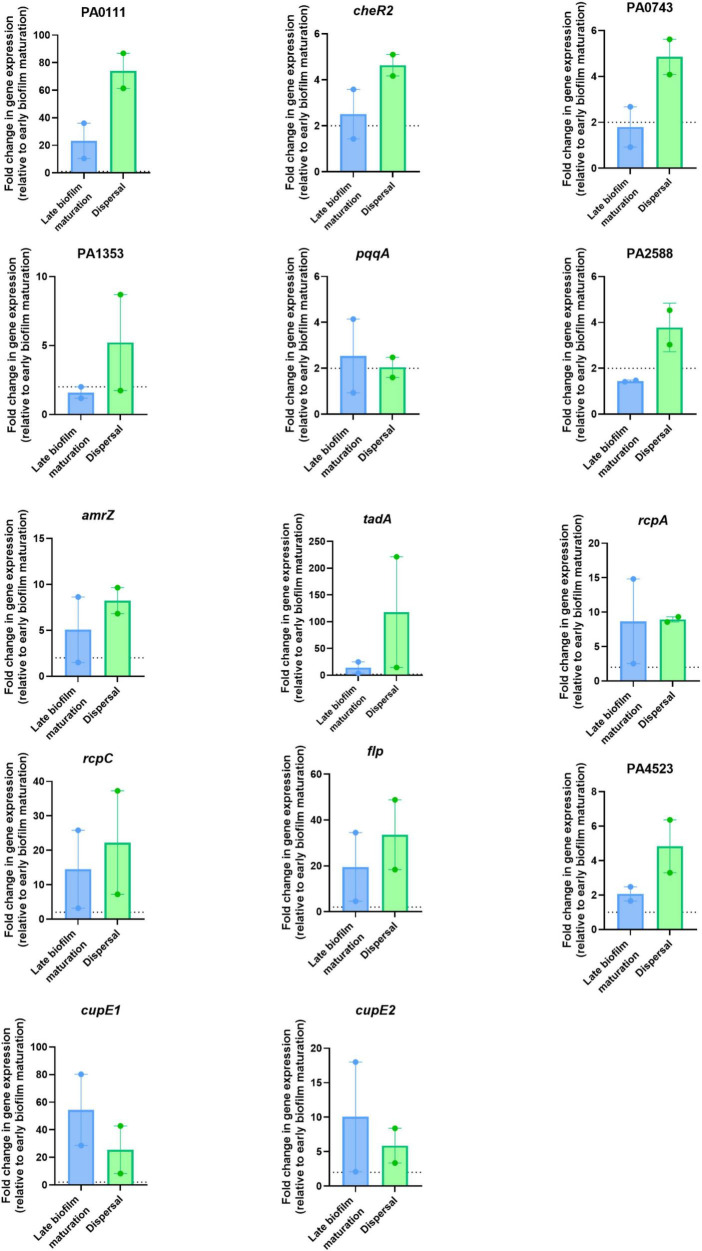
Validation of the transcriptional biomarkers of biofilm dispersal. Differences in transcriptional regulation measured by RT-qPCR for the indicated genes from total RNA samples extracted at 4 h (early biofilm maturation), 8 h (late biofilm maturation) and 12 h (dispersal). Fold-changes at 8 and 12 h are relative to 4 h. Means ± SEM are shown. Two technical replicates are represented.

To evaluate the wider use of these biomarkers beyond the reference PAO1 strain, we investigated gene presence across 1,301 *P. aeruginosa* genomes. Indeed, we identified that across a diverse range of genomic backgrounds, all fourteen transcriptional biomarkers of dispersal are present in ∼100% of complete *P. aeruginosa* genomes available in GenBank, with only *flp* showing an average pairwise identity < 99% (Supplementary Table 2). We restricted our biomarker screen to upregulated transcripts, as elevated expression levels are more consistently detected and validated across analytical platforms. Due to the similarities in transcriptomic profiles between planktonic and biofilm cells collected during dispersal, we hypothesized that biofilm cells initiate the expression of dispersal biomarkers prior to phenotypically manifesting the dispersal response in culture. Therefore, we collected samples from cells undergoing late biofilm maturation and dispersal and validated these biomarker genes by RT-qPCR (Figure 4). Our results confirmed the upregulation of all fourteen genes during dispersal. Furthermore, all genes but four (PA0743, PA1353, *pqqA*, and PA2588) were also significantly upregulated during late biofilm maturation (Figure 4). Genes PA0111, *cheR2*, *tadA*, *rcpC*, *flp*, and PA4523 showed a pattern where transcript abundance increased in mature biofilms (2.5–23-fold) and peaked during dispersal (4.6–117-fold relative to early biofilms) (Figure 4). In contrast, the transcription of *cupE1* and *cupE2* fimbrial genes peaked in mature biofilms (54.41- and 10.1-fold) but sharply declined upon dispersal (25.55- and 5.9-fold) (Figure 4). This transient transcriptional profile suggests that CupE fimbriae genes are likely characteristic of mature biofilms. In contrast, transcription of PA0111, *cheR2, tadA, rcpC, flp* and PA4523 peaked at dispersal (Figure 4). Altogether, these biofilm dispersal biomarkers exhibited strong reproducibility, and their increased transcription prior to the onset of dispersal marks biofilm cells undergoing late biofilm maturation that are ready for an incoming stage transition.

### Reporter plasmids as a low-cost rapid tool for monitoring biofilm dispersal

**FIGURE 5:**
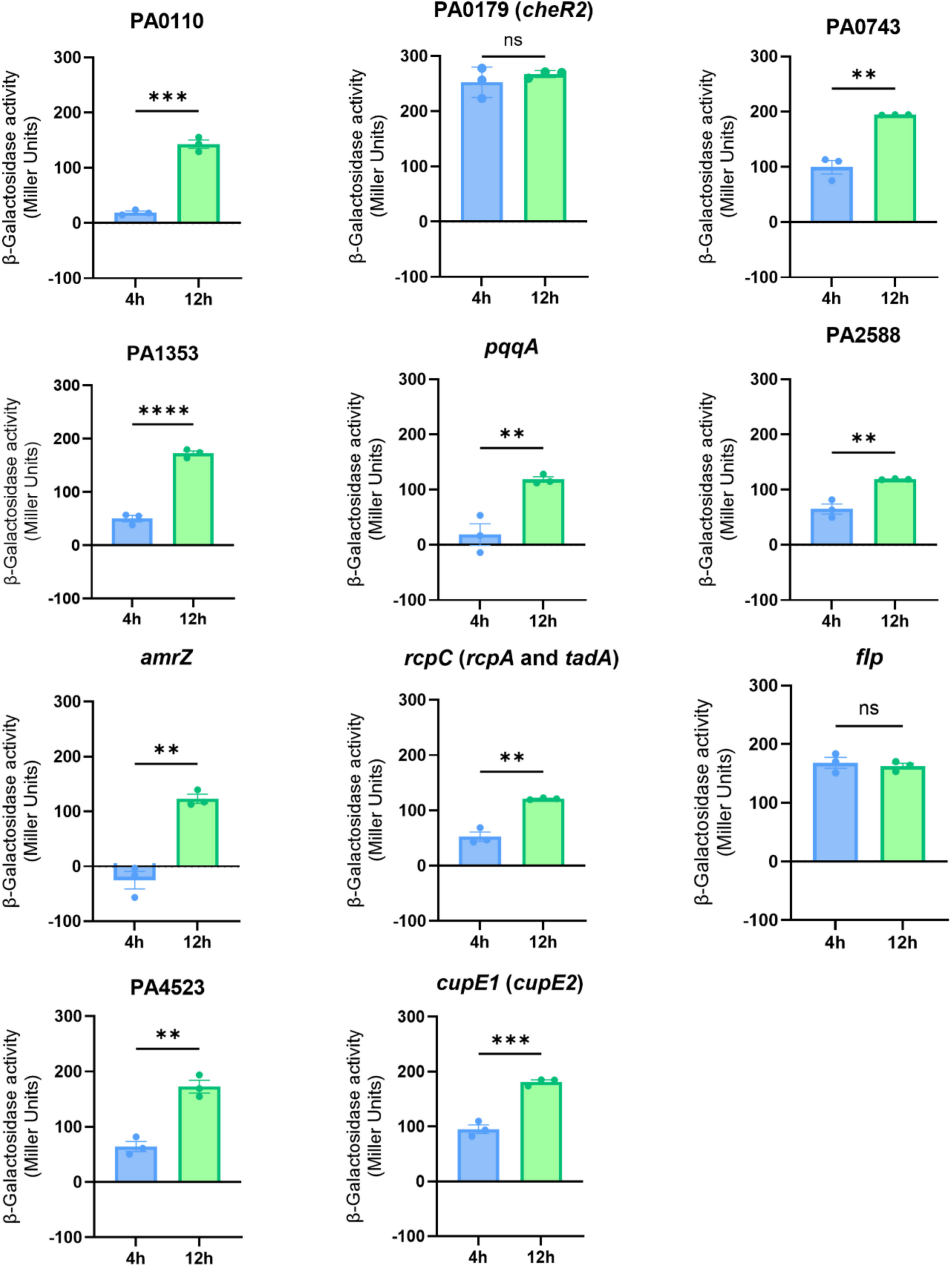
LacZ reporter plasmids indicate the upregulation of the identified biomarkers during dispersal. Differences in transcriptional regulation of biomarkers of dispersal measured by enzymatic activity of β-galactosidase in *P. aeruginosa* PAO1 in biofilm samples extracted at 4 h (early biofilm maturation) and 12 h (dispersal). Means ± SEM are shown. Three technical replicates are represented. Statistical differences between groups were calculated using Student’s *t-*tests (**P < 0.01; ***P < 0.001; ****P < 0.0001). One representative experiment is shown from two independently performed assays.

While RT-qPCR provides a direct and sensitive method to quantify transcriptional differences, we constructed a more scalable reporter system using plasmids carrying *lacZ* transcriptional fusions to the predicted promoter regions of each biomarker, excluding co-operonic genes (*rcpC* also represents *rcpA* and *tadA*, and *cupE1* also represents *cupE2*). To evaluate this approach, *P. aeruginosa* PAO1 strains harboring each reporter plasmid were cultured to early biofilm maturation (4 h) and dispersal (12 h). β-galactosidase activity was significantly increased for 9 of 11 reporters, with only *cheR2* and *flp* showing elevated levels of enzymatic activity at both early biofilm maturation and dispersal (Figure 5). In contrast, reporters containing PA0110, PA0743, PA1353, *pqqA*, PA2588, *amrZ*, *rcpC/rcpA*, PA4523, and *cupE1/cupE2* showed significantly increased enzymatic activity during dispersal relative to early biofilm maturation (Figure 5). Negative enzymatic activity values for *amrZ* at 4 h indicate a very low presence of LacZ, where background noise levels (OD_550_) are similar to total recorded activity (OD_420_; see Methods). Collectively, these results demonstrate that *lacZ* transcriptional reporter plasmids provide a low-cost and rapid detection method for the transcriptional activation of the identified biomarkers of dispersal and further validate these genes as robust biomarkers of the onset of dispersal.

## Discussion

The study of transcriptional changes between each biofilm stage provides valuable insights into the key cellular signaling pathways underlying transitions between sessile and motile lifestyles. Here, we characterized the transcriptional profiles of cells undergoing attachment, biofilm maturation and dispersal, and identified 23 biomarkers characterizing the dispersal response, 14 of which comprising a subset upregulated upon the initiation of dispersal.

Cells undergoing attachment sense and respond to surface contacts through mechanosensory systems such as the Pil-Chp machinery, which relies on type IV pili-mediated twitching motility (Park and Sauer, 2022; Webster et al., 2021). In our study, we report an upregulation of type IV pili biogenesis genes (*pilA, pilB*, and *pilD*) and of the mechanosensory system Pil-Chp (*pilGHIJK-chpABCDE*) during the attachment and biofilm maturation stages compared to dispersing cells. Furthermore, we report the upregulation of *vfr* and *cyaB*, involved in the sequential signal transduction from the Pil-Chp chemotaxis sensor (Park and Sauer, 2022). These were accompanied by the upregulation of *fimX*, which encodes a putative phosphodiesterase essential for the assembly of type IV pili, and of *cupA1* encoding the main subunit of the CupA adhesin (Kazmierczak et al., 2006). Our findings therefore align with previous studies indicating that type IV pili are indispensable to surface-attachment in microplates (Klausen et al., 2003), and are consistent with prior reports that surface-sensing promotes the transcriptional upregulation of genes involved in type IV pilus biogenesis (Cowles and Gitai, 2010).

The intracellular messenger c-di-GMP acts as a master regulator of the bacterial lifestyle, with its accumulation leading to biofilm formation (Rumbaugh and Sauer, 2020). Our dataset revealed the upregulation of several stage-specific genes encoding proteins with validated or hypothetical c-di-GMP-modulating activity. Specifically, *siaD* was the most upregulated during biofilm maturation, which encodes a DGC involved in early microcolony formation and adhesion (Irie et al., 2012; Park and Sauer, 2022). In contrast, biofilm dispersal is associated with increased PDE activity and decreasing intracellular c-di-GMP (Hengge, 2009). This was reflected in our dataset, where 6 genes encoding functionally active PDEs were upregulated. Genes *rbdA* and *nbdA* encode enzymes with well-described roles in biofilm dispersal. Both have been reported to modulate flagellar motility, with RbdA being a key component of the BdlA-regulated core dispersal response (Morgan et al., 2006; Petrova and Sauer, 2012b). Moreover, four upregulated genes (PA1878, PA2572, PA4108, and PA4781) encode PDEs carrying the HD-GYP motif (Stelitano et al., 2013). While the role of these genes in the biofilm dispersal stage is unclear, it has been reported that disruption of PA4108 and PA4781 resulted in higher intracellular c-di-GMP and decreased swarming activity, whereas PA2572 modulated swarming motility (Ryan et al., 2009; Stelitano et al., 2013). We here validated by RNA-seq and RT-qPCR that both PA4108 and PA4781 are strongly upregulated during late biofilm maturation and dispersal, which suggests a potential role in initiating dispersal responses. Additionally, recent reports showed that deletion of *cdpR* led to increased biofilm biomass and decreased swarming motility (Zhao et al., 2016). Considering that the upregulation of *cdpR* is strongly controlled by the quorum sensing regulator RsaL (Zhao et al., 2016), the stage-specific transcriptional activation of *cdpR* would therefore suggest that dispersal in closed systems is mediated by a global quorum sensing-mediated swarming response.

The synthesis of the rhamnolipid biosurfactant is important to the quorum sensing-mediated swarming motility and dispersal (Schooling et al., 2004). In closed culture systems, rhamnolipid was proposed to drive biofilm dispersal (Wood et al., 2018). Here we report that the rhamnolipid and swarming modulator PA2572 together with several genes encoding rhamnolipid biosynthesis genes were highly upregulated during dispersal. Moreover, four of the identified PDEs upregulated during dispersal, including PA4108, PA4781, *nbdA*, and *rbdA*, were previously shown to influence the synthesis of rhamnolipid leading to loss of biofilm biomass (An et al., 2010; Liu et al., 2022; Ryan et al., 2009). Altogether, these data suggest biofilm dispersal in closed systems may be driven by rhamnolipids.

Among the upregulated biomarkers, *amrZ* was reported as a global regulator mediating the upregulation of genes related to alginate biosynthesis and Type IV pili twitching motility while downregulating Pel synthesis, therefore driving biofilm dispersal (Hou et al., 2019; Kalia et al., 2022). In contrast, *tadA*, *rcpA*, *rcpC*, *flp, cupE1*, and *cupE2*, which are regulated under the PprAB two-component system (Bernard et al., 2009; Giraud et al., 2011), were reported to mediate the expression of a highly adherent phenotype (de Bentzmann et al., 2012). The upregulation of *pprB* and their associated regulon has been previously reported to be positively modulated by the quorum sensing signals LasI and RhlI, with evidence suggesting that PprB contributes to the cellular response to RhlI (Lazdunski et al., 2007). Similarly, genes encoding LecA and LecB, two lectins mediating adhesion via recognition of Psl and host sugars, are also regulated by quorum sensing (Diggle et al., 2006; Passos da Silva et al., 2019; Tielker et al., 2005). Considering that our data supports the co-upregulation of quorum-sensing–regulated genes encoding both dispersal factors (e.g., rhamnolipids) and adhesins (type IVb pili, CupE fimbriae and lectins LecA and LecB) (Diggle et al., 2006; Passos da Silva et al., 2019; Tielker et al., 2005), this suggests that quorum sensing may coordinate dispersal while priming cells for re-attachment. This interpretation is consistent with previous studies showing that dispersed cells exhibit an enhanced capacity to re-attach to surfaces (Chambers et al., 2017).

Additionally, we identified PA0110, *cheR2*, *pqqA*, PA0743, PA1353, and PA4523 to be strongly associated with the dispersal stage, which suggests a potential involvement of their gene products in facilitating this process. Their marked upregulation during dispersal warrants further investigation.

Understanding biofilm dispersal may represent a critical step toward combating chronic biofilm-associated infections. As dispersing cells become resensitised to antimicrobial treatment once they are released from the protective biofilm matrix, the underlying transcriptional changes occurring during biofilm dispersal hold promise for unveiling the next generation of antibiofilm treatments and adjuvants. We here report the transcriptional profiles of the *P. aeruginosa* biofilm life cycle, including attachment, biofilm maturation and dispersal stages in closed systems, which can be used as benchmarks for future studies. Additionally, we propose a set of biofilm dispersal biomarkers that are robustly activated not only at the onset of biofilm dispersal, but have been also found upregulated immediately after treatment with the biofilm dispersal agent Spermine-NONOate (Forga et al., 2026). As the upregulation of these biomarkers was consistently reproduced by transcriptomic and RT-qPCR analyses, we generated gene fusion reporters that enable the rapid monitoring of promoter activities *in vivo*, as well as being adaptable to high-throughput (e.g. microplate-based) platforms. Therefore, these reporter plasmids present with potential application in high throughput screens supporting the discovery and development of novel dispersal-inducing antibiofilm therapeutics.

## Materials and methods

### Strains, media, and culture conditions

*Pseudomonas aeruginosa* PAO1 wild-type (WT). Cultures were routinely grown overnight in LB (lysogeny broth) media at 37°C, 200 rpm before incubation in fresh M9 media (9 mM NaCl, 22 mM KH_2_PO_4_, 48 mM Na_2_HPO_4_, 19 mM NH_4_Cl and 2 mM MgSO_4_, 100 μM CaCl_2_, 0.4% glucose, pH 7.0) for each experiment. Where appropriate, cultures were supplemented with 10 μg/mL of gentamicin.

### Biofilm kinetics and microscopy

Biofilms were grown on 24-well plates or tissue culture flasks (Nunc, ThermoFisher Scientific) by inoculating 10^7^ colony forming units (CFU)/ml of overnight bacterial cultures for 2, 4, 8, and 12 h diluted in M9 media. 1 ml of culture was inoculated in each well of microtiter plates and 10 mL were used in tissue culture flasks. Cultures were incubated at 37 *^o^*C and 180 rpm (microtiter plates) or 70 rpm (tissue culture flasks). Shaking speed was adjusted to prevent splashing. At the indicated time points, supernatants were removed, and wells were delicately washed with 1 mL phosphate buffered saline (PBS, Gibco) to remove planktonic cells. Microscopy (PrimoVert, Zeiss) was used to confirm the removal of planktonic cells and preservation of biofilm structures. Remaining biofilm cells were stained with 0.1% (w/v) crystal violet dissolved in 6.25% (v/v) methanol. Stained biofilms were washed twice with PBS and solubilized with ethanol. Biofilm biomass was quantified by measuring the optical density at 550 nm (OD_550_) using a SPECTROStar Nano microplate reader (BMG LabTech). Micrographs of stained biofilms were taken via microscopy as described above.

### RNA sequencing and analysis

Biofilms were cultured and dispersed as previously described with some adjustments (Bertran et al., 2025). Briefly, tissue culture flasks (Nunc, ThermoFisher Scientific) were inoculated with 10^7^ CFU/ml cells in 50 ml of M9 medium from overnight cultures. Cultures were incubated at 37*^o^*C shaking at 70 rpm for 2, 4, or 8 h. At 2 and 8 h, planktonic cells (10^8^) were collected from the liquid phase and immediately mixed with 2 volumes of RNA-protect solution (QIAGEN). Biofilm cells were gently rinsed with 10 ml of PBS and resuspended in 6 mL of RNAprotect (QIAGEN, Cat# 76506) mixed with 3 mL of PBS using cell scrapers. Cells (10^8^) were subsequently pelleted at 5,000 g (10 min, 25^ o^C). RNA was extracted with the RNeasy mini kit (QIAGEN, Cat# 74104) as per the manufacturer’s protocol for Gram negative bacteria grown in minimal media. Extracted RNA samples were treated with DNase I and rRNA was removed using streptavidin-coated magnetic beads (BGI Genomics, Shenzhen, China). Library was prepared by RNA fragmentation and cDNA synthesis followed by the ligation of adaptors at the 3’ end of cDNA fragments. These were further amplified by PCR and single-stranded fragments were circularized for downstream DNBSEQ PE100 sequencing (BGI Genomics, Shenzhen, China). Clean reads were mapped to the *P. aeruginosa* PAO1 reference genome (GenBank Accession number AE004091.2) with Bowtie2 v2.4.5 (Langmead and Salzberg, 2012). Geneious Prime v2024.0.7 was used to generate the read count matrix and to subsequently run DESeq2 v1.50.2 to compare the expression levels between sample groups (attachment, biofilm maturation and dispersal).

The Principal Component Analysis (PCA) plot was conducted exclusively as an exploratory visualization to assess overall transcriptomic similarity within biological replicates and to examine separation between treatment groups. It was generated by extracting the reads per kilobase of transcript per million mapped reads (RPKM) of every gene at each one of the stages (Supplementary Table 1). Then, genes with < | 1| log_2_ fold-change transcription or *P* > 0.01 for all groups were discarded, and the remaining differentially regulated genes were plotted using GraphPad Prism 10.4.1 (Graph Pad, United States).

### Gene sequence conservation analysis

A local database was generated with 1,301 complete *P. aeruginosa* genomes (taxid: 287) were downloaded from GenBank, excluding atypical, metagenome-assembled genomes and genomes from large multi-isolate projects. Gene sequences were extracted from the annotated *P. aeruginosa* PAO1 reference genome AE004091.2. BLAST analyses were performed locally using the NCBI BLAST plugin for Geneious Prime v2024.0.7 against the local *P. aeruginosa* database. In-built Megablast analyses were configured with minimum E value of 10^–8^ and query cover of 75%.

### RT-qPCR

To validate RNA-seq data, total RNA was extracted from cells harvested from different biofilm stages as described above. cDNA was transcribed using 1 μg of total RNA using the SuperScript III first-strand synthesis kit (Invitrogen) as per the manufacturer’s protocol. cDNA samples were then used as templates for RT-qPCR conducted with the Quantinova SYBR Green master mix (Qiagen) as per the manufacturer’s protocol. The housekeeping gene *recA* was used as a control. Each sample was independently assayed twice with the gene-specific primers listed in Supplementary Table 2. Relative gene expression was calculated using the 2^–ΔΔCT^ method (Livak and Schmittgen, 2001). Transcriptional fold changes were plotted using GraphPad Prism.

### Construction of *lacZ* fusion reporter plasmids and β-galactosidase assays

The cloning vector carrying a promoterless *lacZ* was constructed via Gibson Assembly with a 3.2 kb fragment containing *lacZ* from *E. coli* K-12 MG1655, a 150 bp fragment containing the MCS of pBBR1-MCS5 and a 3.8 kb fragment containing a gentamicin resistance cassette and the pBBR1 origin of replication from pBBR1-MCS5, resulting in the reporter construct pBBR1-lacZ. Gene promoter regions were predicted using Sapphire.cnn.pseudomonas (Coppens and Lavigne, 2020). The 5’ untranslated region (∼500 bp) of each gene/operon containing putative promoters were cloned into the MCS between BamHI and HindIII restriction sites upstream of *lacZ*. Plasmid constructs were maintained in *E. coli* DH10-β (10μg/ml of Gentamicin), purified using a Plasmid Mini Kit (Qiagen) following the manufacturer’s protocol and introduced into *P. aeruginosa* PAO1 via electroporation. All constructs and strains involved in this process are summarized in Table 3. A summary of all reporters is provided in Table 3.

**TABLE 3:** Bacterial strains and plasmids used in this study.

Strain or plasmid	Relevant characteristics	Source
Strains		
*P. aeruginosa* PAO1	Wild-type, HER-1018 P1	Holloway (1972)
*E. coli* DH10β	F–*mcr*A Δ(*mrr*-*hsd*RMS-*mcr*BC) φ80*lac*ZΔM15 Δ*lac*X74 *rec*A1 *end*A1 *ara*D139 Δ(*ara-leu*)7697 *gal*U *gal*K λ–*rps*L(StrR) *nup*G	Invitrogen, Cat# 18297010
*E. coli* K-12 MG1655	*E. coli* K-12, Source of *lacZ*	Qin et al. (2023)
Plasmids		
pBBR1-MCS5	Gm^r^, lac promoter, source of MCS5, resistance cassette and ori.	Elzer et al. (1995)
pBBR1-lacZ	Gm^r^, promoterless *lacZ*.	This study
pBBR1-lacZ-PA0110	Gm^r^, *PA0110:*:*lacZ* transcriptional fusion.	This study
pBBR1-lacZ-PA0179	Gm^r^, *PA0179:*:*lacZ* transcriptional fusion.	This study
pBBR1-lacZ-PA0743	Gm^r^, *PA0743:*:*lacZ* transcriptional fusion.	This study
pBBR1-lacZ-PA1353	Gm^r^, *PA1353:*:*lacZ* transcriptional fusion.	This study
pBBR1-lacZ-PA1985	Gm^r^, *PA1985:*:*lacZ* transcriptional fusion.	This study
pBBR1-lacZ-PA2072	Gm^r^, *PA2072:*:*lacZ* transcriptional fusion.	This study
pBBR1-lacZ-PA2588	Gm^r^, *PA2588:*:*lacZ* transcriptional fusion.	This study
pBBR1-lacZ-PA4305	Gm^r^, *PA4305:*:*lacZ* transcriptional fusion.	This study
pBBR1-lacZ-PA4648	Gm^r^, *PA4648:*:*lacZ* transcriptional fusion.	This study
pBBR1-lacZ-*amrZ*	Gm^r^, *amrZ:*:*lacZ* transcriptional fusion.	This study
pBBR1-lacZ-*nicD*	Gm^r^, *nicD:*:*lacZ* transcriptional fusion.	This study
pBBR1-lacZ-*nbdA*	Gm^r^, *nbdA:*:*lacZ* transcriptional fusion.	This study
pBBR1-lacZ-*flp*	Gm^rrr^, *flp:*:*lacZ* transcriptional fusion.	This study

β-galactosidase-based reporter assays were performed as previously described (Miller, 1972). Briefly, 10^8^ cells were collected by centrifugation from biofilm phases at early biofilm maturation or biofilm dispersal stages and OD_600_ was recorded. Cells were lysed with 1 mg/mL lysozyme in TE buffer (10 mM Tris-HCl, 1 mM EDTA). Cell lysates were resuspended in 1 mL of Z-buffer (0.06 M Na_2_HPO_4_, 0.04 M NaH_2_PO_4_, 0.01 M KCl, 0.001 M MgSO_4,_ 0.05 M β-mercaptoethanol) and 160 μL were transferred to a 96-well plate in triplicate (Nunc, ThermoFisher Scientific). Then, 40 μL of an ONPG solution (4 mg/mL) in phosphate buffer (0.06M Na_2_HPO_4_, 0.04 M NaH_2_PO_4_; pH 7.0) were added to each well and reactions were incubated for 2 h at 28 *^o^*C in a plate reader. Absorbances were recorded at 420 and 550 nm every 10 min and Miller units (MU) were calculated using the following equation:


$$M\,U=1000\,x\,\frac{\mathrm{OD420}-1.75*\mathrm{OD550}}{t*v*\mathrm{OD600}}$$

Where *t* is the reaction time, *v* is the volume of culture used and OD600 is the density of the culture prior to centrifuging.

Statistical differences in enzymatic activity between cells collected at early biofilm maturation or biofilm dispersal were calculated using Student’s *t*-tests (**P < 0.01; ***P < 0.001; ****P < 0.0001). One representative experiment is shown from two independently performed assays.

## Data Availability

The RNA-Seq data have been deposited in the NCBI Short Read Archive (SRA) database with accession code PRJNA1291829.
